# Utilization of continuous professional development among health professionals in East Ethiopia: a multi-health facility-based cross-sectional study

**DOI:** 10.1186/s12909-024-05036-7

**Published:** 2024-01-12

**Authors:** Tesfaye Assebe Yadeta, Ahmed Mohammed, Adisu Alemu, Kerimo Behir, Bikila Balis, Shiferaw Letta

**Affiliations:** 1 School of Nursing and Midwifery, College of Health and Medical Sciences, Haramaya University, P.O. Box. 235, Harar, Ethiopia; 2 School of Medicine, College of Health and Medical Sciences, Haramaya University, Harar, Ethiopia; 3 School of Public Health, College of Health and Medical Sciences, Haramaya University, Harar, Ethiopia

**Keywords:** Continuous professional development, Utilization, Barriers, Health professionals, East Ethiopia

## Abstract

**Background:**

Healthcare workers must maintain their knowledge, attitude, and skills regarding the most recent technology and competencies to deliver quality health care. The Ministry of Health, Ethiopia developed guidelines and directives for the utilization of continuous professional development programs. However, there is limited evidence on utilization and barriers to utilization in the study area. Therefore, this study aimed to assess the utilization and barriers to the utilization of continuous professional development among health professionals working in health facilities in eastern Ethiopia.

**Methods:**

A health facility-based cross-sectional quantitative study was conducted among 731 healthcare professionals from September 01, 2022, and October 30, 2022. A multistage sampling technique was utilized. A simple random sampling technique selected health facilities and study participants. A self-administered questionnaire developed from national continuous professional development guidelines was disseminated to healthcare professionals working in the selected forty health centers and four hospitals. STATA statistical package version 14 was used for data analysis. A descriptive summary was used to summarize the variables. A logistic regression model was used to assess the association between independent variables and the outcome variable. Adjusted odds ratios along with 95% CIs were estimated to assess the strength of the association, and a *p*-value < 0.05 was used to declare the level of statistical significance in the analysis.

**Results:**

Continuous professional development utilization was determined for 731 healthcare professionals, of whom 241 (32.97%) [(95% CI: (29.55, 36.38)] had utilized continuous professional development. Lack of continuous professional development knowledge AOR 0.23 [(95% CI: 0.14, 0.37)], being female AOR 0.58 [(95% CI: 0.39, 0.86)], lack of internet access AOR 0.62 [(95% CI: 0.43, 0.89)], greater than 20 km distance from main road AOR 0.58 [(95% CI: 0.37, 0.91)], not heard importance of continuous educational units AOR 0.45 [(95% CI: 0.31, 0.65)], and poor perceived need of continuous professional development AOR 0.61 [(95% CI: 0.38, 0.97)], had a negative statistically significant association with the utilization of continuous professional development.

**Conclusion:**

The utilization of continuous professional development in the study area was low. Health sectors and stakeholders working on continuous professional development programs are required to focus on developing strategies for knowledge creation, female health workers, and access to the internet, remote areas, information on the importance of continuous educational units, and the variety of needs of professionals for continuous professional development implementation.

## Background

Continuing Professional Development (CPD) is a range of learning activities through which health professionals maintain and develop their throughout their careers to ensure that they retain their capacity to practice safely, effectively, and legally within their scope of practice [1]. CPD aims to sustain and develop the competencies of health professionals, which is crucial in response to the diverse health service needs of the community, newly emerging or re-emerging health problems, and scientific development [2, 3]. Previous studies have shown that CPD intervention improves professional practice, knowledge, attitude, compliance with desired practice, and behavioral change [4, 5]. Other benefits include improved communication skills [6], improved self-confidence and self-esteem, career progression [7, 8], and better workforce retention [9]. As such CPD is effective in improving the healthcare system, patient outcomes, and community quality health services [10, 11].

Implementation of CPD programs in low-resource settings like ours is still challenging [12]. The utilization of CPD is low and variable, especially in low-income countries [13]. Only about 21.7% of World Health Organization Afro-region member states’ nurses and midwives complete mandatory CPD requirements [14]. In Sri Lanka 78.2% [15], in Jordan only 44.8% [16], and in Kenya 58.6% [17] were involved in CPD activities. Studies showed that limited availability [18], lack of time, insufficient clinical coverage, lack of funding, remoteness, personal behavior [19], availability of accredited CPD courses, absence of privileges in an institution [2], lack of opportunity, frequent rotating to different departments, personnel turnover [20], cost and access to digital resources or internet [5, 21] were factors affecting the utilization of CPD.

Continuing professional development implementation is one of the prioritized national strategic plans, expected to improve quality health care and health outcomes of the community [3]. The Ministry of Health (MOH), Ethiopia recognized the significance of CPD in its policies and strategic plans in 2013 and updated guidelines and implementation directives addressing standardization, regulation, and accreditation mechanisms in 2018 [1]. The establishment and accreditation of CPD providers and accreditors began in 2020 [22]. Continuing professional development guidelines in Ethiopia mandate that no person shall practice as a health professional without having a professional practice license issued by the regulatory bodies. The professional practice license given to any health professional shall be renewed every three years upon ethical and competence evaluation [23].

Healthcare workers in Ethiopia can obtain continuous professional units (CEUs) by engaging in CPD activities such as training, research, modules, guidelines, and course development, participation in scientific conferences, guest/occasional lecturers at accredited institutions, workshops, moderating panel discussions, brief communication, structured health education sessions, educational visits, and case reports. The training modality can be face-to-face, online, or blended [2]. Study findings suggest that multistrategy modality approaches to deliver CPD positively influence the effectiveness of CPD intervention strategies [19]. The MOH gives authority and responsibility to develop courses and process the courses' accreditation by accreditors for the CPD providers. The CPD providers request training costs from trainees or organization sponsors. These expose trainees to costs like training fees, transportation, internet services, and accommodation for hotels [23].

Although CPD implementation is one of the priority areas for the national strategic plan to improve the health system in Ethiopia, there is limited information on utilization and barriers to utilization of CPD in Ethiopia. Therefore, this study aims to assess the utilization and barriers to the utilization of CPD among healthcare workers in eastern Ethiopia.

## Methods and materials

### Study area and period

The study was conducted among health professionals working in health facilities found in the East Hararghe Zone, Oromia Regional State, eastern Ethiopia, from September 01, 2022, and October 30, 2022. This Zone is bordered on the southwest by Bale, on the west-by-West Hararghe Zone, on the north by Dire Dawa Administration, and on the north and east by the Somali Region. The East Hararghe zone has twenty districts, three towns, eight hospitals, and 121 health centers. Health professional human resource coverage is 40.6%. Currently, there are three CPD providers that serve the Harari regional state and the East and West Harahghe zones are available [22].

### Study design

A health facility-based cross-sectional quantitative study design was done.

### Study population

All healthcare professionals (medicine, nurse, pharmacy, medical laboratory, midwifery, and public health) working in the health facilities found in Eastern Ethiopia were the sources of population. Healthcare professionals randomly selected from the selected health facilities were the study population. Environmental health workers since MOH did not include them at the time of the CPD policy implementation and there were no approved CPD courses for them, health professionals on annual and maternity leave, critically ill, and not available at health facilities during data collection were excluded.

### Sample size determination and sampling procedures

The sample size was determined by using a single proportion population with an expected frequency of 50%, a confidence level of 95%, a confidence limit of 5%, and a design effect of two. The final sample size was 768. Therefore, 768 health professionals were randomly selected for inclusion in the study. A multistage sampling technique was employed. The total number of health facilities and lists of health professionals obtained from the Zonal health bureau. A total of 121 health centers and eight hospitals found in the East Hararghe Zone, 40 health centers, and four hospitals were selected by a simple random sampling technique. Study participants were selected by simple random sampling technique from the selected health facilities. For each health facility, the sample size was allocated based on the proportion of their health professional load.

### Data collection

A structured questionnaire was developed to collect relevant data after reviewing related works of different literature. The questionnaire contains questions related to socio-demographic variables, CPD related knowledge and perception, and utilization of CPD. The CPD related questions were developed from the National Continuing Professional Development Guidelines [2]. Data were collected using pretested questionnaires. The pretested questionnaire was done on 38 (5%) of the sample size of healthcare professionals in health facilities who were not included in actual data collection. The amendment to the questionnaires was made based on the findings. A self-administered data collection method was employed. Ten BSc health professionals collected the data. Five MSc/MPH holder health professionals are involved in supervision. The data collectors and supervisors took two days of intensive training before the actual work about the aim of the study, procedures, data collection techniques, the art of interviewing, and ways of collecting the data. The data collectors distributed the questionnaires to the selected healthcare workers and collected the questionnaire after checking its completeness. The supervisors performed intensive supervisions, and then checked the data for completeness, accuracy, and consistency throughout the data collection period. The overall supervision was performed by principal investigators and co-investigators. Finally, double data entry was performed by two data clerks, and data validation was done.

### Variables and measurements

#### Dependent variable is utilization of CPD

Utilization of CPD refers to an individual obtaining CEU at least once in participation in any CPD activities recommended by national CPD guidelines (training, research activities, modules, guidelines, courses developed, scientific conferences, guest/occasional lecturers at an accredited institution, workshops, moderating panel discussions, brief communication, structured health education sessions, educational visits, case reports). Continuous professional development utilization was documented based on professionals' replies and labeled as “yes” and “no” and coded with 1 and 0, respectively.

#### Independent variables include

Socio-demographic, and health facility-related characteristics, knowledge of CPD, Perceived CPD needs, Reason for non-Utilization, and Preferences for CPD utilization. The age of the health professionals was documented based on professionals' replies and later grouped as ≤ 25, 26–35, and 36 + with codes 1, 2, and 3, respectively for analysis. Marital status was grouped as “married” and all others as “others” and was coded 0 and 1, respectively. Years of services were documented based on professionals' replies and later grouped as ≤ 5, 6–10, and 10 + with codes 1, 2, and 3, respectively for analysis. Distance from the main road was documented based on professionals' replies and later grouped as ≤ 20 km and 20 + km with codes 1, and 2, respectively for analysis. Knowledge of CPD and access to the internet were documented based on professionals' replies and labeled as “yes” and “no” and coded with 1 and 0, respectively.

#### Perceived CPD needs

Perceived need refers to an individual's judgment about the necessity or benefits of CPD. The question assesses seven areas (clinical, management and leadership, communication, teaching/coaching, research, and ethics) of training needs, which scores each of 0–4 for every seven areas of need: “0” need no at all, “1” neutral, “2” moderate need, “3” need, and “4” need most. Scoring was performed by counting the number of boxes checked in a column. Multiply that number by the value indicated above, and then add the subtotal to produce a total score. The total score for the seven items ranges from 0 to 28. Scores of 0–13, and 14–28 represent poor perceived need, and good perceived need, respectively.

### Data analysis

All filled questionnaires were checked for completeness and consistency, and double data entry was made using Epidata 3.1 software. The data were exported to the STATA statistical package version 14 for further analysis. Descriptive statistics were used to summarize the data. A logistic regression model was used to assess the association between independent variables and the outcome variable. Multicollinearity among independent variables was tested before entering them into the multivariable model using the variance inflation factor test, the tolerance test, and values of the standard error. Model fitness was tested by Hosmer–Lemeshow goodness-of-fit tests. Crude odds ratios with 95% CIs were estimated to assess the association between each independent variable and the outcome variable. Variables with a *p*-value ≤ 0.2 in the bivariate analysis were considered in the multivariable analysis by assuming that the outcome varied according to risk factors. The adjusted odds ratio (AOR) along with 95% CIs was estimated to assess the strength of the association, and a *p*-value < 0.05 was used to declare the level of statistical significance.

## Results

### Socio-demographic and health facility-related characteristics

The study enrolled a total of 768 healthcare professionals. Of these, 731 of them were responded, making a response rate of 95.18%. The age of participants ranged from 19 to 58 years with a mean (standard deviation) of 28.11 (± 5.78) years. About two-third of 464 (63.47%) participants were males, 341 (46.65%) had five or fewer years of working experience, and 308 (42.13%) had no internet access (Table 1).

**Table 1:** Socio-demographic characteristics of health professionals working in East Hararghe Zone health facilities, Eastern Ethiopia, 2022 (*n* = 731)

Variables		Frequency	Percentage
Health facilities	Hospital	284	38.85
	Health center	447	61.15
Age	≤ 25	246	33.65
	26–35	435	59.51
	≥ 36	50	6.84
Sex	Male	464	63.47
	Female	267	36.53
Marital status	Married	470	64.30
	Others	261	35.70
Years of services	5 and less	341	46.65
	6–10	272	37.21
	> 10	118	16.14
Field of study	Nursing	344	47.06
	Midwifery	127	17.37
	Pharmacy	110	15.05
	Medical laboratory	60	8.21
	Public health	53	7.25
	Medicine	37	5.06
Level of education	Diploma	356	48.70
	Bachelor degree	363	49.66
	Second degree	12	1.64
Availability of internet	Yes	423	57.87
	No	308	42.13
Distance from main road	≤ 20 km	122	16.69
	> 20 km	609	83.31

### Utilization of CPD and reason for non-utilization

**Fig. 1:**
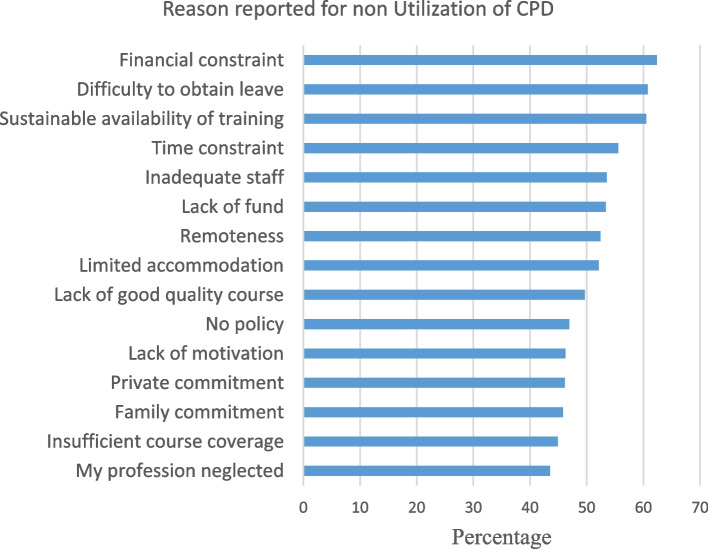
Reasons reported for the no utilization of CPD among health professionals working in health facilities in the East Hararghe Zone, Eastern Ethiopia, 2022

Of all 731 study participants, 32.97% (95% CI: 29.55%, 36.38%) utilized CPD and obtained CEU. The majority of them, 163 (69.07%), were obtained by face-to-face training, 159 (67.37%) through online training, and only 23 (9.74%) of them were utilized from workshops. However, the participants did not utilize other recommended methods of utilization of CEU, such as research, conference, guideline review and development, course development, panel discussion, brief communication, health education, case development, and case reports. The main reason reported for not utilizing CPD was financial constraints (62.38%). Difficulty obtaining leave (60.74%) and sustainable availability of training (60.47%) were also frequent reasons mentioned by the participants (Fig. 1).

### Preferences for CPD utilization

**Fig. 2:**
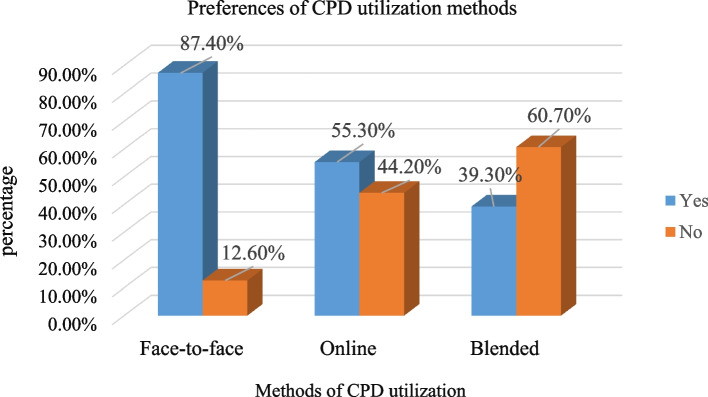
Preferences of CPD utilization methods by health professionals working in health facilities in East Hararghe Zone, Eastern Ethiopia, 2022

Healthcare workers were asked about their preferences for CPD utilization methods, and the majority of them preferred face-to-face (87.41%) training (Fig. 2).

### Predictors of Utilization of CPD

In bivariate logistic analysis, being female, lacking internet access, having health facilities distance greater than 20 km from the main road, lacking CPD knowledge, having no hearing of the importance of CEU, and having poor perceived need for CPD showed statistically significant associations with utilization of CPD. To estimate the relative contribution of each factor to the utilization of CPD, AOR was derived from logistic regression models. Lack of CPD knowledge was associated with a 77% [AOR = 0.23 (95% CI: 0.14, 0.37)] reduction in the utilization of CPD. Similarly, the odds of CPD utilization among females was 42% [AOR = 0.58 (95% CI: 0.39, 0.86)] lowered than the odds of male health workers. Lack of internet access was associated with a 0.38% [AOR = 0.62 (95% CI: 0.43, 0.89%)] reduction in the utilization of CPD compared to those who had internet access. The odds of utilizing CPD was 42% [AOR = 0.58 (95% CI: 0.37, 0.91)] reduced in health facilities greater than 20-km distance from the main road compared to their counterparts, heard no CEU associated with a 55% [AOR 0.45, (95% CI: 0.31, 0.65)] reduced in the utilization of CPD. Poor perception of the need for CPD was associated with a 0.39% [AOR = 0.61(95% CI: 0.38, 0.97)] reduction in the utilization of CPD compared to those who had a good perception of the need for CPD (Table 2).

**Table 2:** Results of bivariate and multivariable logistic regression models on factors associated with CPD utilization among healthcare workers in the East Hararghe Zone, Eastern Ethiopia, 2022

Variables	Utilization of CPD *n* = 731		Crudes OR		Adjusted OR	
	No (n/%)	Yes (n/%)	COR	95% CI	AOR	95% CI
Age in year						
≤ 25	173 (70.33)	73 (29.67)	1		1	
26–35	283 (65.06)	152 (34.94)	1.27	0.90, 1.78	1.13	0.73, 1.75
≥ 36	34 (68.00)	16 (32.00)	1.11	0.57, 2.14	1.24	0.53, 2.86
Sex						
Male	279 (60.13)	185 (39.87)	1		1	
Female	211 (79.03)	56 (20.97)	0.40	0.28, 0.56*	0.58	0.39, 0.86*
Marital status						
Married	312 (66.38)	158 (33.62)	1		1	
Other	178 (68.20)	83 (31.80)	0.92	0.66, 1.27	0.85	0.56, 1.27
Years of services						
≤ 5 years	231 (67.74)	110 (32.26)	1		1	
6–10 years	181 (66.54)	91 (33.46)	1.05	0.75, 1.48	0.86	0.56, 1.32
> 10 years	78 (66.10)	40 (33.90)	1.07	0.69, 1.67	0.96	0.53, 2.72
Access of internet						
Yes	260 (61.47)	163 (38.53)	1		1	
No	230 (74.68)	78 (25.32)	0.54	0.39, 0.74*	0.62	0.43, 0.89*
Distance from main road						
≤ 20 km	71 (58.20)	51 (41.80)	1		1	
> 20 km	419 (68.80)	190 (31.20)	0.63	0.42, 0.94*	0.58	0.37, 0.91*
Know CPD						
Yes	267 (55.51)	214 (44.49)				
No	223 (89.20)	27 (10.80)	0.15	0.09, 0.23	0.23	0.14, 0.37*
Heard importance of CEU						
Yes	144 (48.98)	150 (51.02)	1		1	
No	346 (79.18)	91 (20.82)	0.25	0.18, 0.34*	0.45	0.31, 0.65*
Perceived need of CPD						
Good	373 (64.20)	208 (35.80)	1		1	
Poor	117 (78.00)	33 (22.00)	0.50	0.33, 0.77*	0.61	0.38, 0.97*

Note: "*" shows a statistically significant association of utilization of CPD with independent variables along with 95% CIs, and a *p*-value < 0.05

## Discussion

In this study, CPD utilization among health professionals was 32.97%. Factors that were significantly associated with less CPD utilization were CPD knowledge, sex, internet access, distance from the zone, awareness of the importance of CEU, and perceived need for CPD.

The CPD utilization reported in this study was low, indicating that the majority of health professionals have not participated in CPD. This finding is consistent with those reported in studies from low-income countries [24]. However, it was lower than those reported from high-income countries where guidelines and policies for CPD utilization were available for more than 20 years [25]. In Ethiopia, approximately 219 CPD providers and only 37 CPD accreditors were established. Of the 219 CPD providers, 132 (62.27) and 13 (35.13%) CPD accreditors were found in the capital city Addis Ababa [22]. Inadequate numbers, uneven regional distribution, and slow progress for implementation since the 2013 recognition of the limited capacity of health workers negatively affect the quality of healthcare delivery and patient outcomes [22].

In this study, we found that female health workers utilized CPD less than male health workers. In low-income countries such as Ethiopia, female health workers focus on activities at home, such as giving care to their children and feeding their families [26]. Moreover, women often face challenges in accessing and participating in CPD activities, inadequate digital skills, and access to digital resources, resulting in low utilization of CPD [27]. Tackling this issue requires a collaborative effort from CPD providers, employers, government organizations, and civil society groups to create a more inclusive environment that encourages women's participation. In addition, providing options such as remote learning or flexible schedules, raising awareness about gender disparities within CPD, and fostering a supportive environment is crucial. Encouraging husbands to take on more responsibilities within the family was also helpful [28].

The availability of the Internet has a great contribution to CPD activities, and health professionals can participate online [29, 30]. There are many accredited courses online developed even in the country [22]. In this study, we found that participants who had no internet access had lower utilization of CPD, which may be due to poor access to online courses or lack of information about CPD. Poor ICT infrastructure, internet connectivity, and digital skills retarded the use of digital learning technology for CPD [30].

Distance from the main road was also reported to lower the utilization of CPD among health professionals. This finding is consistent with a study in Ireland showing that distance to CPD events affects CPD utilization [31]. Similarly, study in Mexico showed CPD engagement remote area is low and challenging [24]. Distance from the main road or remoteness of the health facilities area may be related to cost and lack of access to information [30].

In this study, we found that health workers who had no knowledge of CPD and never heard of the importance of CEU utilized CPD less, which is consistent with the findings of studies conducted elsewhere [13, 31]. Knowledge helps health professionals in actual practice [32], informs decision making [33] and is an asset for action [34, 35]. In this study, the participant obtained CEU only through a few recommended modalities, such as face-to-face training, online training, and workshops, which may indicate a lack of knowledge of other modalities.

In this study, we found that the perceived need for CPD was associated with the utilization of CPD. This finding is consistent with a study conducted in South Africa that found that the perception of the importance of continuing professional development prepares health professionals for the utilization of CPD and that it enhances the ability to meet public needs [36]. Studies showed HCW-identified CPD needs and current programs reduce enthusiasm for CPD [37]. This suggested that healthcare workers must realize that CPD is their responsibility.

Our findings also suggest that multiple modalities take advantage of CPD utilization. In addition to face-to-face and online learning, blended learning enhance the opportunities. Blended CPD implementation includes a variety of learning approaches, such as in-person, accessing guidelines and print, social media, instructor-led, teamwork, peer-to-peer interaction, self-study, and individual work [38].

### Strength and limitation

The study covers multiple health facilities and a large area. The tool was developed from CPD national guidelines. The primary limitation of cross-sectional studies is that the temporal link between the outcome and the exposure cannot be determined because both are examined at the same time.

## Conclusion

The utilization of continuous professional development in the study area was low. Health sectors and stakeholders working on continuous professional development programs are required to focus on developing strategies for knowledge creation, female health workers, and access to the internet, remote areas, information on the importance of continuous educational units, and the variety of needs of professionals for continuous professional development implementation.

## Data Availability

The data sets used for this study are available from the corresponding authors upon reasonable request.

## Abbreviations

AOR: Adjusted Odds Ratio
CEU: Continuous educational Unit
CI: Confidence intervals
COR: Cruds Odds ratio
CPD: Continuous Professional Development
FMOH: Federal Minster of Health
WHO: World Health Organization
